# Structural, Magnetic and Microwave Characterization of Polycrystalline Z-Type Sr_3_Co_2_Fe_24_O_41_ Hexaferrite

**DOI:** 10.3390/ma13102355

**Published:** 2020-05-20

**Authors:** Svetoslav Kolev, Petya Peneva, Kiril Krezhov, Tanya Malakova, Chavdar Ghelev, Tatyana Koutzarova, Daniela Kovacheva, Benedicte Vertruyen, Raphael Closset, Lan Maria Tran, Andrzej Zaleski

**Affiliations:** 1 Institute of Electronics, Bulgarian Academy of Sciences, 72 Tsarigradsko Chaussee, 1784 Sofia, Bulgaria; petya_venelinova_peneva@abv.bg (P.P.); kiril.krezhov@gmail.com (K.K.); tanya_malakova@abv.bg (T.M.); chavdarghelev@yahoo.com (C.G.); tatyana_koutzarova@yahoo.com (T.K.); 2 Institute of General and Inorganic Chemistry, Bulgarian Academy of Sciences, Academy Georgi Bonchev Street, bld. 11, 1113 Sofia, Bulgaria; dkovacheva@gmail.com; 3 Greenmat, Chemistry Department, University of Liege, 11 allée du 6 août, 4000 Liège, Belgium; b.vertruyen@uliege.be (B.V.); Raphael.Closset@uliege.be (R.C.); 4 Institute of Low Temperature and Structure Research, Polish Academy of Sciences, Ul. Okólna 2, 50-422 Wroclaw, Poland; l.m.tran@intibs.pl (L.M.T.); a.zaleski@intibs.pl (A.Z.)

**Keywords:** Z-type hexaferrite, magnetic phase transition, microwave properties, reflection losses

## Abstract

We report results on the structural and microwave properties and magnetic phase transitions in polycrystalline Sr_3_Co_2_Fe_24_O_41_ hexaferrite synthesized by sol-gel auto-combustion and acting as a filler in a composite microwave absorbing material. The zero-field-cooled (ZFC) and field-cooled (FC) magnetization curves revealed a change in the magnetization behavior at 293 K. The reflection losses in the 1–20 GHz range of the Sr_3_Co_2_Fe_24_O_41_ powder dispersed homogeneously in a polymer matrix of silicon rubber were investigated in both the absence and presence of a magnetic field. In the latter case, a dramatic rise in the attenuation was observed. The microwave reflection losses reached the maximum value of 32.63 dB at 17.29 GHz in the Ku-band. The sensitivity of the microwave properties of the composite material to the external magnetic field was manifested by the appearance of new reflection losses maxima. At a fixed thickness *t_m_* of the composite, the attenuation peak frequency can be adjusted to a certain value either by changing the filling density or by applying an external magnetic field.

## 1. Introduction

In recent years, intensive efforts have been focused on the development of optimized electronic equipment, such as antennas, modems, mobile phones, magnetic recorders, etc., operating in the microwave, mainly gigahertz, range. Operation at such frequencies may encounter serious problems arising when the transmission/emission of electromagnetic (EM) signals from an unwanted source is jamming through electromagnetic interference (EMI) the electronic devices functioning in a similar frequency range [1]. This has provoked increased interest in both basic and applied research in the field of microwave absorbing materials [2,3,4,5,6].

To absorb or reduce the reflection of microwave radiation, various materials, such as ferrous alloys, metallic materials, and magnetic materials, are widely used. Among the most commonly used magnetic materials are the ferrites, which are structurally classified as spinels, garnets and hexaferrites. In general, hexaferrites are ferromagnetic materials, with their magnetic properties closely correlated to their crystalline structure classified as M, Y, W, Z, X and U type [7,8]. They are characterized by a well-expressed magneto-crystalline anisotropy, or, the magnetization produced has a preferred orientation within the crystal structure. Thus, they can be distinguished as belonging to two basic groups: those with an easy axis of magnetization, or uniaxial hexaferrites, and those with an easy plane or cone of magnetization, referred to as ferroxplana or hexaplana materials. Besides other attractive properties, they exhibit a high resonance frequency and good magnetic permeability and electrical resistivity. The resonance frequency can be as high as 100 GHz, which explains why they are being researched in view of fabrication of microwave absorbers [9,10].

The attention to the Z-type hexaferrites in particular has been stimulated by possibilities for applications such as radio-frequency and microwave components for the fast-growing industry of multifunctional mobile devices [5,11]. For example, Ba_3_Co_2_Fe_24_O_41_ (Co_2_Z) is a good candidate for use in GHz antennas in the ultra-high frequency band, as it was shown by Lee et al. [6]. It is also one of the most promising microwave-absorbing ferrites because of the favorable magnetic properties resulting from the *c*-plane anisotropy at higher frequencies. It has a saturation magnetization of 51 emu/g and Curie temperature (*T_c_*) of 680 K. Co_2_Z type ferrites have a static magnetic permeability of 12–15, with a resonance frequency of 1.5 GHz [12,13,14,15]. The combination of the magneto-electric effect at room temperature with appropriate high-frequency electromagnetic characteristics further enhances the interest in studying the properties of Z-type hexaferrites. For the first time, the existence of a significant ME effect in hexaferrites at room temperature was reported by Kitagawa et al. [16]; particularly, the Z-type hexaferrite Sr_3_Co_2_Fe_24_O_41_ with spiral magnetic structure was found to exhibit a low-field (~10 mT) magnetoelectric effect at room temperature. Many efforts have also been directed to improving the high-frequency resonance and reflectivity properties of Sr_3_Co_2_Fe_24_O_41_.

It is well known that the Z-type hexaferrites have a very complex crystal structure, being a combination of M-type and Y-type hexaferrites [17]. Their unit cell consists of S-, R- and T-blocks. The structure can be described as alternating stacking of the basic blocks RSTSR*S*T*S* (the asterisk corresponding to a block rotation by 180° around the hexagonal c-axis), where S is (Ba,Sr)Fe_2_O_4_, R is [(Ba,Sr)Fe_6_O_11_]_2_ and T is (Ba,Sr)_2_Fe_8_O_14_. The number of atoms in the unit cell is 140, which is manifested by the relatively long *c* length—51.91 Å [18].

The complexity of this crystal structure is among the primary reasons placing hidden obstacles to the easy preparation of this type of hexaferrites. The small difference in the temperatures of phase transitions from one to the other type of the large hexaferrite family is one such complication, which additionally hinders the preparation of single-phase samples, so that secondary phases of W and U-hexaferrites [19,20] often remain in the end product. Therefore, in order to obtain samples with homogeneous composition, techniques other than the solid-state reactions routes, such as those of soft chemistry, are needed. One such technique is the sol-gel auto-combustion, which has often proved its merits in the successful synthesis of hexaferrites [21]. In this paper, we report on the structural, magnetic and microwave properties of a Sr_3_Co_2_Fe_24_O_41_ material synthesized by sol-gel auto-combustion with sugar as a fuel, and on its use as a filler in a polymer matrix to form a composite performing as a microwave antireflection material.

## 2. Materials and Methods

The Sr_3_Co_2_Fe_24_O_41_ powders were synthesized by sol-gel auto-combustion. The metal nitrates Sr(NO_3_)_2_, Co(NO_3_)_2_, 6H_2_O and Fe(NO_3_)_3_. 9H_2_O were used as starting materials and sugar was used as a fuel. The metal nitrates were dissolved in distilled water and the obtained solution was homogenized for six hours. A sugar-containing solution was added to the metal cation solution thus prepared and homogenized for 18 h. The solution was slowly evaporated (120–130 °C) to form a gel. During the dehydration process, the gel turned into a fluffy mass and burned in a self-propagating combustion manner. During the auto-combustion process, the burning gel volume expanded rapidly and NO*_x_* gas resulting from the nitrate ions decomposition was released. The material produced was ground and annealed at 600 °C at a heating rate of 200 °C/h. The powder was cooled slowly to room temperature at an average cooling rate of 70 °C/h. The resulting precursor material was homogenized by vibration milling, pressed into disk-shaped pellets with a diameter of 16 mm, and synthesized at 1200 °C for seven hours. The samples were quenched rapidly to room temperature. The pellets were crushed and ground, after which the powder was pelletized in the shape of disks with a diameter of 16 mm. Then the pellets were synthesized at 1250 °C for three hours, and finally quenched rapidly to room temperature to prevent the formation of other hexaferrite phases. X-ray diffraction (XRD) measurements were conducted to assess the phase purity; these also yielded distinct features concerning the unit cell parameters’ variation. Finally, the material was ground and sieved to produce the final hexaferrite powder to be used for magnetic and microwave measurements.

The hexaferrite materials (powder and disk-shaped pellets) were characterized by XRD for phase identification and for assessment of the phase purity using a Bruker D8 Advance Twin diffractometer. The microstructure of the samples was investigated by scanning electron microscopy (Philips ESEM XL30 FEG), while the magnetic properties were measured by using a SQUID Quantum Design magnetometer. The hysteresis measurements were conducted at 4.2 K and at room temperature. The zero-field-cooled (ZFC) and field-cooled (FC) magnetization-vs.-temperature (4.2–300 K) measurements were performed in a magnetic field of 100 Oe.

The microwave measurements were performed on a composite sample prepared as a mixture of the final hexaferrite powder and commercial silicon rubber (Mastersil, ASP) as the polymer matrix. An appropriate amount of hexaferrite powder was homogeneously dispersed in the polymer matrix by mechanical stirring at 200 rpm for 15 min at room temperature to form composite samples. Three composite samples with different filler amounts (per 1 cm^3^ of silicon rubber) were prepared: A (1.8 g/cm^3^), B (2.4 g/cm^3^) and C (3.0 g/cm^3^). The samples were molded into a toroidal shape with an outside diameter of 7 mm, an inner diameter of 3 mm and a thickness of 4 mm, with the filler concentration in weight % being increased at an equal step from sample A to sample C. A referent sample (denoted R) of silicon rubber only was also prepared in a toroidal shape to study the polymer matrix influence on the microwave properties.

The microwave (MW) measurements were conducted using a Hewlett-Packard 8756A microwave scalar network analyzer in the frequency range 1–20 GHz. To determine the MW characteristics of the composites we employed a technique whereby the electromagnetic wave (TEM) impinges normally on a single-layer absorber backed by a perfect conductor [22,23]. The prepared toroidal samples were tightly fit into a 50 Ω coaxial measurement cell (APC 7) backed by a perfect conductor (short-circuit measurement). A calibration was performed prior to the experiment. The attenuation of the reflected wave was measured using an air-filled sample holder, with the results showing *R_L_* = 0 dB. This is a fast and precise technique allowing one to measure directly the reflection losses, *R_L_* [dB], of the sample studied. During these measurements, an external magnetic field was applied using a permanent magnet providing a flux density of 1.4 T. The magnetic force lines were perpendicular to the direction of electromagnetic wave propagation. The magnetic flux density in the air gap in the coaxial line was 0.3 T, as measured by a Model 475 Gaussmeter with an HMNT-4E04-VR Hall sensor.

## 3. Results and Discussion

**Figure 1:**
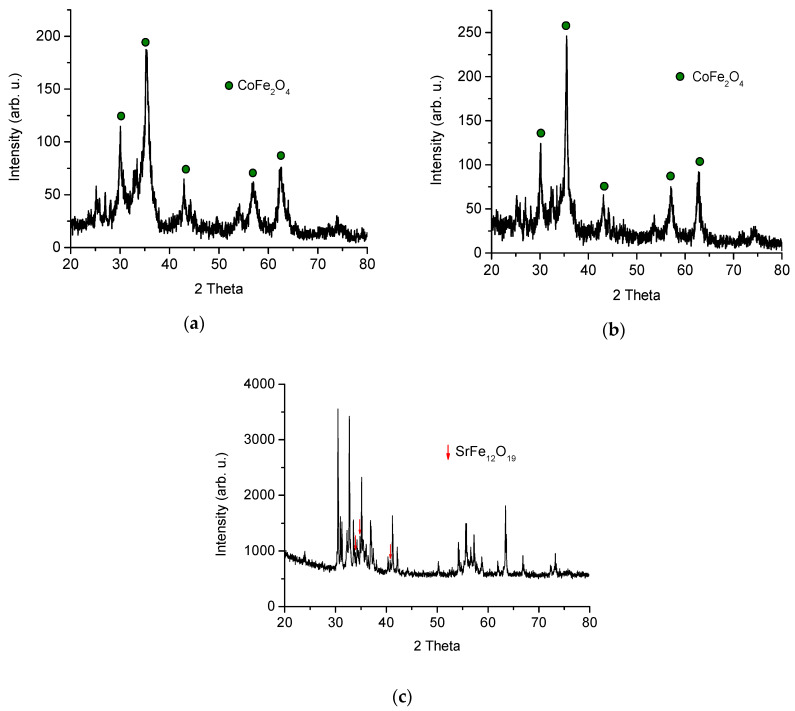
X-ray diffraction (XRD) patterns of Sr_3_Co_2_Fe_24_O_41_: (**a**) as-prepared powder after auto-combustion, (**b**) powder annealed at 600 °C and (**c**) bulk material after three hours at 1250 °C and quenching.

Unlike the preparation of M-type hexaferrites ((Ba,Sr)Fe_12_O_19_) by “wet chemistry”, the complexity of the Z-type hexaferrite structure imposes the need of a progressive transformation through intermediate ferrites before achieving the final structure. It is well known that the synthesis of Z-type hexaferrites often ends with the presence of other types of hexaferrites. Figure 1 illustrates the different stages of the process of synthesis by sol-gel auto-combustion. Figure 1a shows that the auto-combusted material was not well crystallized; moreover, peaks of the CoFe_2_O_4_ spinel ferrite were detected. The CoFe_2_O_4_ phase is consistent with the standard pattern JCPDS: 00-022-1086. The sample exhibits a slightly higher crystallization degree after the heat treatment at 600 °C (Figure 1b). The XRD pattern (Figure 1c) of the powder material used for magnetic and microwave measurements shows the characteristic peaks corresponding to Sr_3_Co_2_Fe_24_O_41_ as the main phase; traces of a minority phase SrFe_12_O_19_ (JCPDS: 00-033-1340) were also observed. However, as explained below, this minority phase did not play a significant role in determining the magnetic and microwave properties observed.

**Figure 2:**
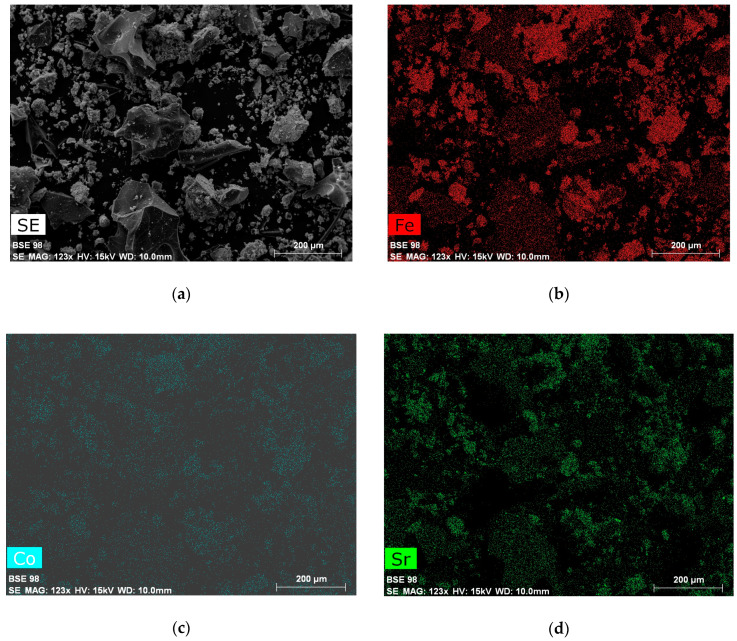
SEM image of the powder after auto-combustion (**a**) and color maps of the elements (Fe-red (**b**), Co-blue (**c**) and Sr-green (**d**)).

**Figure 3:**
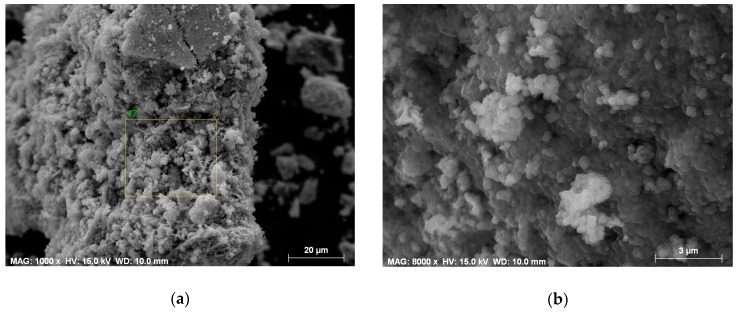

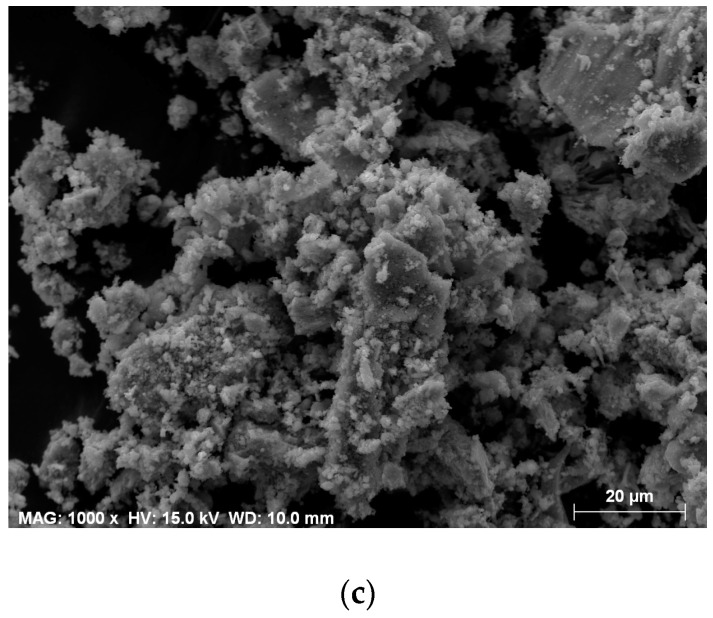
SEM images of the auto-combusted sample (**a**) energy-dispersive X-ray (EDX) analysis area, (**b**) the particles after auto-combustion and (**c**) the sample annealed at 600 °C.

Figure 2 shows a typical morphology of the samples following the auto-combustion process; the elemental analysis mapping shows that all elements are distributed homogeneously in the powder prepared. The energy-dispersive X-ray analysis (EDX) of the auto-combustion sample (Figure 3a) shows that the Sr:Co:Fe ratio is 3:2.1:23.9, which corresponds to the empirical formula Sr_3_Co_2_Fe_24_O_41_. Figure 3b exhibits the presence of spherical particles with a particle size of 150 nm immediately after the completion of the auto-combustion process. We assume that they are of CoFe_2_O_4_, as it is the only crystalline phase after the auto-combustion process. Figure 3c demonstrates the morphology of the precursor powder after heating at 600 °C.

**Figure 4:**
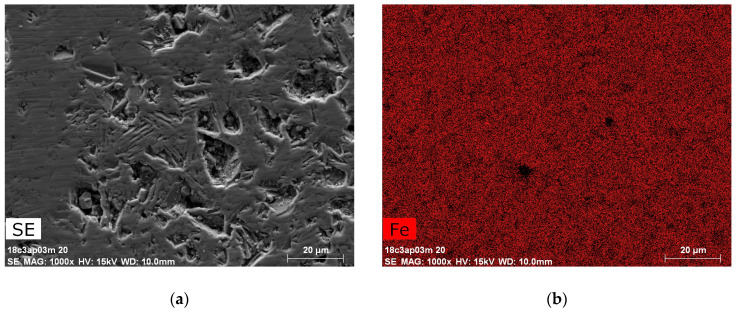

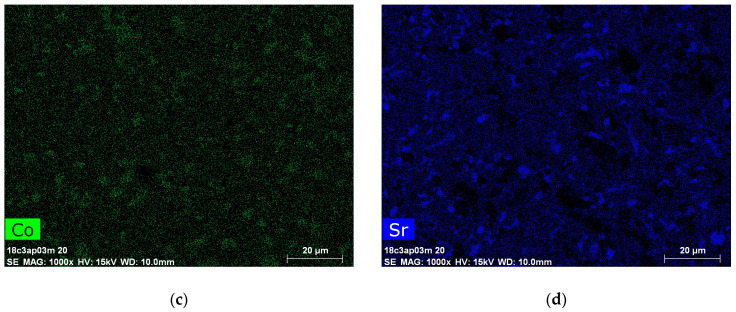
SEM image of cross-section of bulk Sr_3_Co_2_Fe_24_O_41_ (**a**) and color maps of the elements (Fe-red (**b**), Co-green (**c**) and Sr-blue (**d**)).

**Figure 5:**
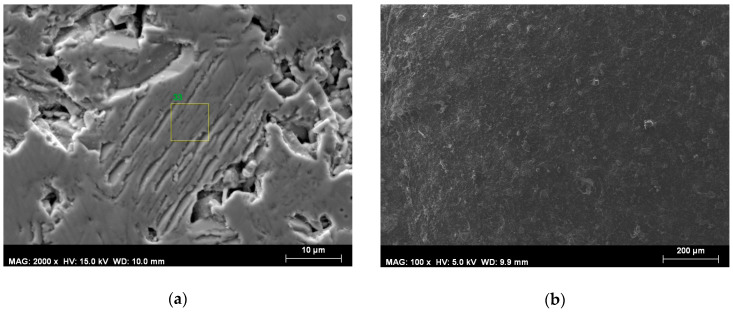
SEM image of (**a**) cross-section of bulk Sr_3_Co_2_Fe_24_O_41_ and (**b**) cross-section of composite sample B.

Figure 4 and Figure 5a show a typical morphology of a cross-section of a bulk pellet; the elemental analysis mapping demonstrates that the elements are distributed homogeneously. Large areas are observed of packages of hexagonal sheets where the separate hexagonal particles are ordered along the *c* axis. The EDX data of these areas (Figure 4b–d) showed that the Sr:Co:Fe ratio was 3:2.01:24.2, corresponding to the stoichiometric one in Sr_3_Co_2_Fe_24_O_41_. Figure 5b presents a typical morphology observed for all composite materials. As can be seen, the magnetic particles are homogeneously dispersed in the polymer matrix.

**Figure 6:**
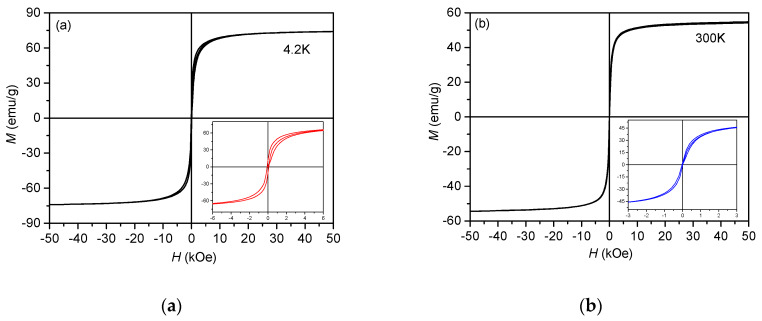
Hysteresis curves of Sr_3_Co_2_Fe_24_O_41_ powder material at 4.2 K (**a**) and 300 K (**b**).

Figure 6 presents the hysteresis loops of the powder material measured at 300 K and 4.2 K. The magnetic parameters, namely, the magnetization (*M_s_*) at 50 kOe, the remanent magnetization (*M_r_*) and the coercive field (*H_c_*) obtained from the curves are listed in Table 1. The hysteresis curves are typical for soft magnetic materials, such as the Z-type hexaferrites. At sufficiently high magnetic fields (>25 kOe), the initial magnetic curves tend to saturation. The *M* values measured at a magnetic field of 50 kOe were 74.01 emu/g at 4.2 K and 54.77 emu/g at 300 K. These values are in good agreement with the ones reported previously [24]. The values of the coercive field and remanent magnetization are very low, which is characteristic for the magnetically soft materials. We emphasize this fact, since it is well known that SrFe_12_O_19_ is a hard magnetic material; thus, the hysteresis curves present evidence that the small amount of residual strontium hexaferrite did not affect the magnetic properties of our samples.

**Figure 7:**
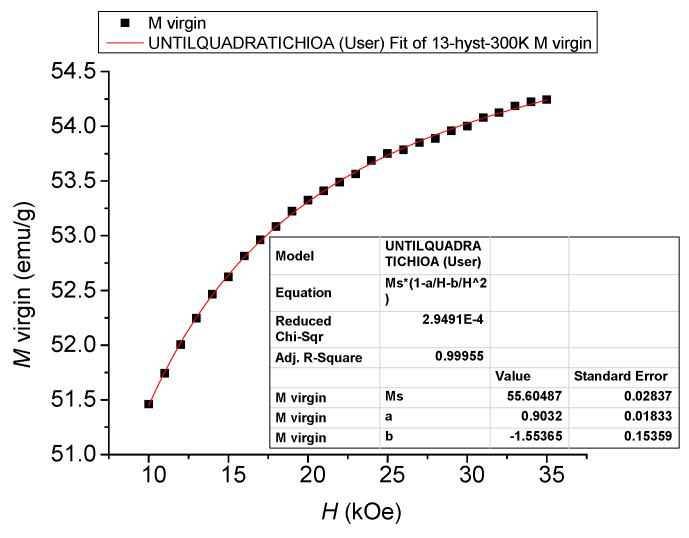
Representative fitted curve for *M* at 300 K calculated by the law of approach to saturation.

For the hysteresis loop at 300 K, the value of the saturation magnetization *M_s_* was calculated using the law of approach to saturation [25] in the field range 10 kOe–30 kOe; a representative fitted curve of *M_s_* thus calculated is shown in Figure 7.

(1) $$M=M_{S}\left(1-\frac{a}{H}-\frac{b}{H^{2}}\right)+\chi H$$

The fitting parameters *M_s_*, *a*, *b* and *χ* have the following meanings: *M_s_* is the saturation magnetization, *a* is the inhomogeneity constant, the term *b*/*H*^2^ is related to the magneto-crystalline anisotropy, and *χ* is the high-field differential susceptibility.

The anisotropy field, *H_a_*, was calculated with the fitting parameter *b* as defined above in the case of a hexagonal symmetry from the relation below [21,25]:

(2) $$b=\frac{H_{a}^{2}}{15}$$

Analyzing the measured magnetization curves according to Equation (1), we found that the coefficient *b* is negative. This effect has already been observed for other compounds [26] and fundamentally explained by the demagnetizing effect of the internal inhomogeneities of the material [27]. The calculated value for *H_a_* at 300 K is 4.82 kOe.

**Figure 8:**
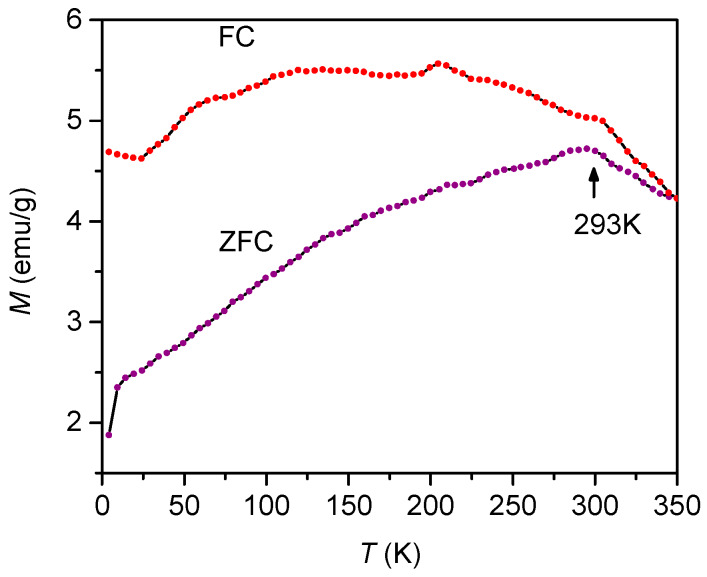
Zero-field-cooled (ZFC) and field-cooled (FC) magnetization measurements of Sr_3_Co_2_Fe_24_O_41_ powder in a magnetic field of 100 Oe.

The ZFC and FC magnetization measurements of the Sr_3_Co_2_Fe_24_O_41_ powder in a magnetic field of 100 Oe are presented in Figure 8. The maximum at 293 K on the ZFC-curve indicates a magnetic phase transition. Following the powder neutron diffraction result of Takada et al. [28], the magnetization anomaly near room temperature is due to the spin arrangement changing from ferrimagnetic to transverse conical spin order and is likely to facilitate the magnetic-field-induced electric polarization at least up to 300 K.

**Figure 9:**
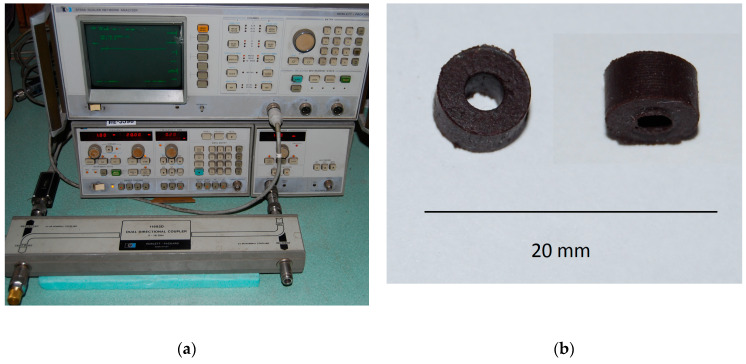
Photographs of the experimental setup (**a**) and of a toroidal sample-two points of view (**b**).

The final step of our study was to measure the microwave characteristics of the powder material dispersed in a polymer matrix (silicon rubber). Photographs of the experimental setup and of a toroidal sample are shown on Figure 9.

Figure 9a presents the frequency dependence of the reflection losses (*R_L_*) of composite samples with different weight-percent filling of the matrix (samples A, B, C) and of the control polymer sample without filler (R) with the same thickness (4 mm) (see Table 2).

Silicon rubber is transparent to electromagnetic waves in the microwave region 1–20 GHz [29,30], as confirmed in Figure 9 for the control (R); thus, in our case of a hexaferrite/silicon rubber composite, the microwave properties are due to the hexaferrite only. The curves presenting *R_L_* were obtained under the assumption that the electromagnetic wave is incident perpendicularly to the surface of the toroidal samples backed by a perfect conductor. Based on the transmission line theory, *R_L_* as a function of the normalized input impedance can be given by [31]:

(3) $$R_{L}\left(dB\right)=20\log\left|\frac{Z_{in}-Z_{0}}{Z_{in}+Z_{0}}\right|$$

where

(4) $$Z_{in}=Z_{0}\sqrt{\frac{\mu_{r}}{\varepsilon_{r}}}\tanh\left[j\frac{2\pi }{c}\sqrt{\mu_{r}\varepsilon_{r}}fd\right]$$

The electromagnetic wave incident on a metal-backed layer sample causes a partial reflection from the air-absorber interface and a partial reflection from the absorber-metal interface. When the characteristic impedance of free space is matched with the input characteristic impedance of an absorber, *Z_in_* = *Z*_0_, impedance matching condition occurs. In addition, the electromagnetic energy can be absorbed completely and dissipated into heat through magnetic and dielectric losses. The two reflected waves might be out of phase by 180° at a certain frequency and can cancel out. The cancellation happens when the thickness of the absorber layer satisfies the quarter-wave thickness criterion described by the quarter-wave theory [32,33]:

(5) $$t_{m}=\frac{nc}{4f_{m}\sqrt{\left|\varepsilon_{r}\mu_{r}\right|}}\left(n=1,3,5,\ldots \right)$$

and

(6) $$f_{m}=\frac{nc}{4t_{m}\sqrt{\left|\varepsilon_{r}\mu_{r}\right|}}\left(n=1,3,5,\ldots \right)$$

where *t_m_* and *f_m_* are the matching thickness and the peak frequency [34].

**Figure 10:**
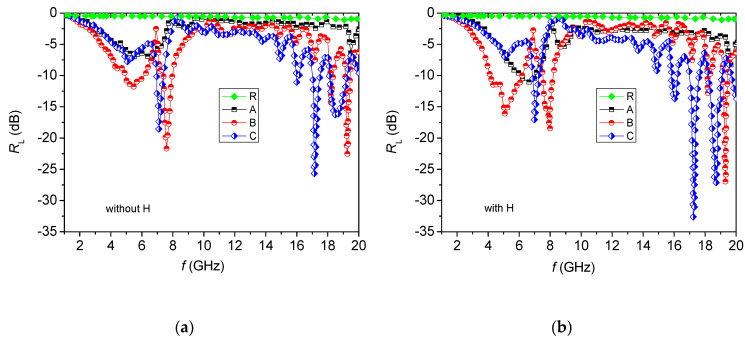
Reflection losses of samples with different filler ratio: (**a**) without magnetic field, (**b**) with an external magnetic field applied.

Achieving superior electromagnetic wave absorption properties necessitates that two important factors be taken into account. One prerequisite is fulfilling the impedance matching condition between the electromagnetic wave absorber and free space. To achieve zero reflection at the front surface of the samples, the characteristic impedance of the specimens ought to be equal/close to that of free space. Reaching a good impedance matching requires making a material with the same or similar *ε_r_* and *μ_r_*. The second significant parameter is the microwave attenuation within the absorber, which could be sufficient to dissipate the propagating microwaves by intrinsic magnetic and/or dielectric loss processes. [35] The minimum of reflection losses corresponds to a minimal reflection or to a MW energy absorption for a given density of filling at the respective frequency–matching frequency (Figure 10).

There exist two matching areas—in the regions of 4–9 GHz and 13–19 GHz. The first matching frequency is located in the magnetic resonance region [36], while the second matching frequency has its origin in a quarter-wavelength resonator [37].

**Figure 11:**
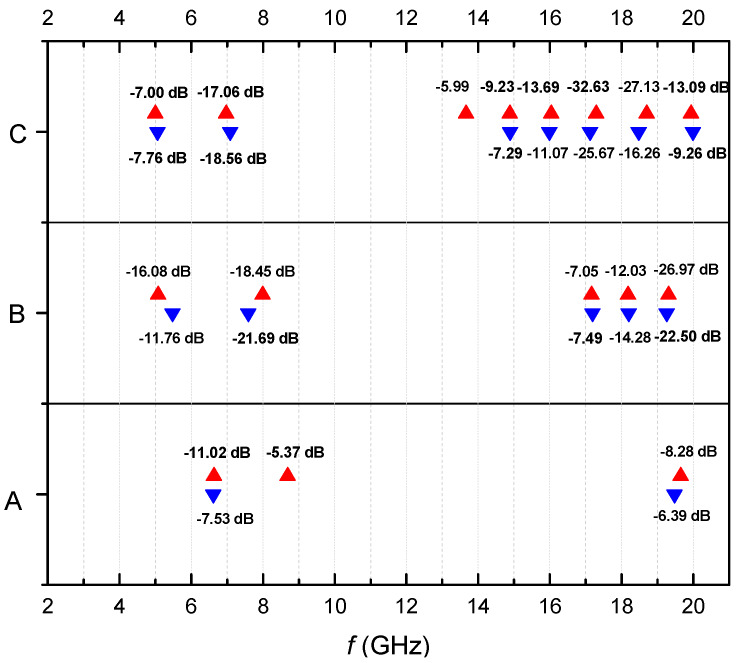
The main peaks (<−5 dB) of the reflection losses for samples A, B and C vs. the frequency.

The reflection losses depend on increasing the density of filling in a specific manner. For sample A, the value of the reflection losses is the lowest—the reflection loss is −7.53 dB at 6.62 GHz. As the filling density is increased in the region 4–9 GHz, the deepest and broadest peaks are observed for sample B (Figure 9a). For sample C, well pronounced peaks appear in the 13–19 GHz band. The same behavior is observed in the case of an applied external magnetic field (Figure 10b). The distribution of the main peaks (<−5 dB) of the reflection losses for samples A, B and C at the respective frequencies with (marked in red) and without (marked in blue) applied external magnetic field is presented in Figure 11. It is seen that most of the peaks existing prior to applying the field become deeper once the magnetic field is applied, as the pairs overlap almost perfectly.

According to Equations (4)–(6), the matching frequency depends on the sample thickness and on its impedance; the latter can be varied by changing the density of filling or the filler’s electromagnetic anisotropy. In this regard, we examined the influence of the external magnetic field on the microwave properties of the composite samples.

Thus, for sample A, the maximum *R_L_* of −7.53 dB was observed at 6.62 GHz without, and *R_L_* of −11.53 dB at 6.64 GHz, with a magnetic field. For this sample, the external magnetic field increases the losses, although they remain rather low (below −15 dB).

For sample B, there were two peaks with *R_L_* larger than −15 dB—at 7.59 GHz and 19.26 GHz, with *R_L_* = −21.69 dB and *R_L_* = −22.5 dB, respectively. After applying the magnetic field, three peaks were seen with *R_L_* larger than −15 dB, namely, at 5.08 GHz, 7.99 GHz and 19.31 GHz with *R_L_* = −16.08 dB, *R_L_* = −18.45 dB and *R_L_* = −26.97 dB, respectively.

Three strong peaks of reflection losses at 7.09 GHz, 17.12 GHz and 18.48 GHz were observed for sample C, with *R_L_* values of −18.56 dB, −25.67 dB and −16.26 dB, respectively. The external magnetic field changed the peaks of reflection losses as follows: to 6.98 GHz, 17.29 GHz and 18.7 GHz with *R_L_* values of −17.06 dB, −32.63 dB and −27.13 dB, respectively (Figure 11).

**Figure 12:**
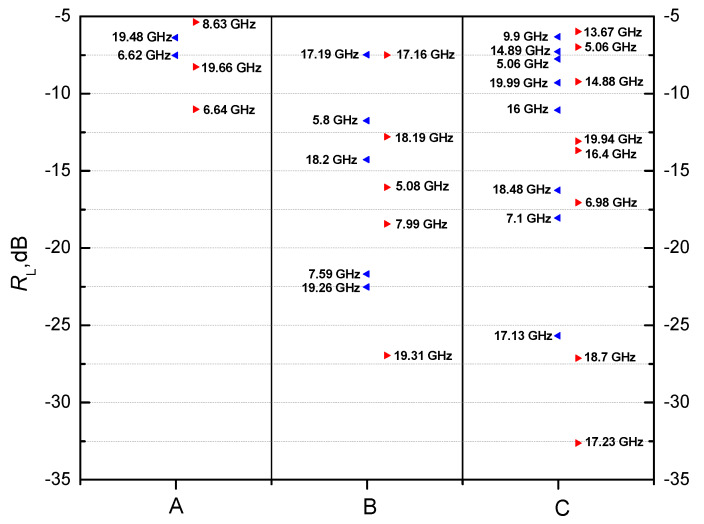
The main peaks (<−5 dB) of the reflection losses *R_L_* for samples A, B and C.

A significant attenuation with the magnetic field applied was observed for all samples A, B and C. The highest attenuation value was measured in the range 17–20 GHz. Thus, the presence of a magnetic field led to a significant increase of the reflection loss peaks for all samples, as is shown in Figure 12 (marked in red).

According to Wu et al., a magnetic field of 0.3 T was sufficient to induce a magnetic phase transition and the appearance of electric polarization [38]. As estimated by other researchers, the dielectric constant at room temperature and 1 GHz changed by approximately 16% in a magnetic field of only 0.032 T [39]. In our case, the magnetic field of 0.3 T applied normally to the composite material induced a change of the magnetic spin orientation and the appearance of electric polarization. The reflection losses are also attributed to the changed dielectric constant and magnetic permeability.

**Figure 13:**
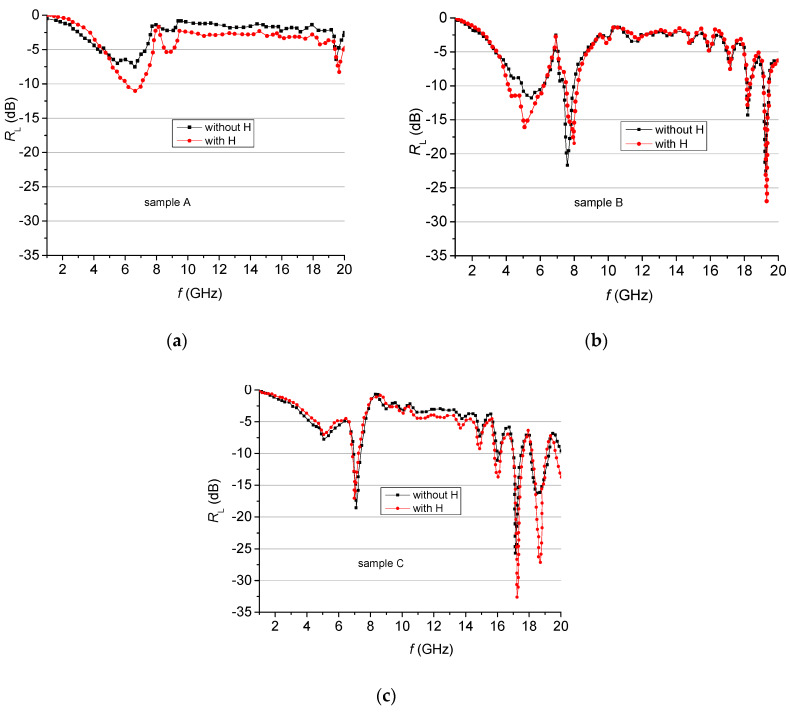
Reflection losses for samples (**a**–**c**) with and without an external magnetic field.

Applying an external magnetic field deepens the reflection losses peaks for all samples (Figure 13). The increased value of *R_L_* is due to the changed *ε_r_* and *μ_r_* according to Equations (3) and (4) as the external magnetic field is applied. Therefore, the frequency of attenuation peaks of composite samples with a fixed thickness *t_m_* can be controlled easily by changing the filling density or by applying an external magnetic field, so that these materials can be used as antireflection coatings or absorbers at a specific frequency. Briefly, by increasing the concentration of the magnetic component and/or by applying a magnetic field, we changed the impedance of the composite sample, which changed the matching frequencies and the magnitude of the reflection losses observed.

## 4. Conclusions

Sr_3_Co_2_Fe_24_O_41_ powder material containing large regions with spontaneously magnetized areas of hexagonal sheets ordered along the *c*-axis was prepared by sol-gel auto-combustion, characterized, and used as a filler material in a polymer matrix. A magnetic phase transition was found to occur at 293 K, in the range of temperatures where a noteworthy low-field magnetoelectric effect in Z-type hexaferrites had been recently reported. The microwave characteristics of the composite structures were addressed in the 1–20 GHz range by measuring the reflection losses, which were found to be strongly affected by a magnetic field. We observed zero reflection at certain frequencies, the matching frequencies, for which the incident and reflected waves of composite specimens are out of phase by 180° and, as a result, the waves on the interface air-sample surface are completely canceled. In order to achieve zero reflection on the front surface of the samples, by applying an external magnetic field we controlled the characteristic impedance of the samples to become close to that of free space; we thus obtained good impedance matching, as similar *ε_r_* and *μ_r_* were reached. The magnetically-induced increase in the reflection loss is explained through the changed electromagnetic thickness, induced electromagnetic anisotropy by the spin arrangement changing from ferrimagnetic to transverse conical spin order, and the magnetic-field induced electric polarization of the composite structures, which lead to changes in the dielectric constant and magnetic permeability. The result was the significant deepening of the majority of the existing peaks, as the maximum reflection loss achieved was 32.63 dB at 17.29 GHz.

## Figures and Tables

**Table 1:** Magnetic properties of Sr_3_Co_2_Fe_24_O_41_ powder material.

*T* (K)	*M_s_* at 50 kOe (emu g^−1^)	*M_r_* (emu g^−1^)	*H_c_* (Oe)
300	54.77	2.38	30
4.2	74.01	11.61	92

**Table 2:** Composite samples with different weight-percent filling of the matrix.

	Sample A	Sample B	Sample C	Sample R
hexaferrite powder, g	1.8	2.4	3.0	0
silicone rubber, cm^3^	1	1	1	1
